# Assessment of left ventricular function by two-dimensional speckle-tracking echocardiography in small breed dogs with hyperadrenocorticism

**DOI:** 10.1186/s13028-014-0088-5

**Published:** 2014-12-31

**Authors:** Hung-Yin Chen, Yu-Hsin Lien, Hui-Pi Huang

**Affiliations:** Institute of Veterinary Clinical Science, Veterinary School, National Taiwan University, No. 1, Section 4, Roosevelt Road, Taipei, 106 Taiwan; Azu Clinic for Animals, No. 92, Section 1, Kin-Shan South Road, Taipei, 100 Taiwan

**Keywords:** Canine, Left ventricular deformation, Hypercortisolism, Hypertension, Hypertrophic cardiomyopathy

## Abstract

**Background:**

Hyperadrenocorticism (HAC) is associated with an increased prevalence of hypertension. This study investigated the left ventricular function using two-dimensional speckle-tracking echocardiography (2D-STE) in small breed dogs affected with spontaneous HAC.

Age-matched healthy controls (*n* = 9), dogs with pituitary-dependent hyperadrenocorticism (PDH, *n* = 10), and dogs with adrenal-dependent hyperadrenocorticism (ADH, *n* = 9) were included in this study. Conventional echocardiography, global longitudinal and circumferential strain, and strain rate were assessed.

**Results:**

On group-wise comparison, left ventricular free wall (LVFWd) and interventricular septal thickness in diastole (IVSd) were thickest in the ADH group, followed by the PDH and controls (*P* = 0.014 and *P* = 0.001, respectively). Neither LVFWd nor IVSd was correlated with systemic blood pressure (*P* = 0.238 and *P* = 0.113, respectively). The values of all variables derived from the global strain and strain rate in longitudinal and circumferential directions followed the same pattern: highest in the controls, followed by PDH and then ADH (all *P* < 0.05, respectively). On multiple regression analyses, global longitudinal strain, global longitudinal strain rate in systole and early diastole, and global circumferential strain all decreased linearly with increased IVSd (all *P* < 0.05).

**Conclusions:**

Left ventricular hypertrophy (LVH) was more prevalent in the HAC group compared to the control group. Association between hypertension and development of LVH was not identified. Decreased global longitudinal and circumferential strains were associated with increased IVSd. 2D-STE revealed significant decreases in systolic functions that were undetected using conventional echocardiography in the ADH and PDH groups.

## Background

Hyperadrenocorticism (HAC) is a condition of chronically elevated circulating glucocorticoid concentration. In response to stimulation of the renin-angiotensin system and mineralocorticoid and glucocorticoid receptors of myocytes, development of systemic hypertension, left ventricular hypertrophy (LVH), and myocardial fibrosis are commonly observed in human patients with HAC [1-3]. The left ventricular wall thickness is significantly increased in HAC patients with concomitant hypertension compared with non-HAC hypertensive controls. Increased ventricular wall thickness is also detected in normotensive patients with HAC compared with age-, sex- and body mass index-matched controls [4]. Active HAC increases risk of myocardial fibrosis compared to both normal subjects and hypertensive patients with similar left ventricular wall thickness and mass index [5]. These findings indicate that hypercortisolism exacerbates changes in ventricular mass.

Human patients with HAC and concomitant hypertension have a two-fold greater risk of detection of cardiac mass abnormality compared with normotensive patients and hypertensive controls [4]. Hypertension may also lead to LVH, which is characterized by concentric remodeling [6-8]. Systemic hypertension is also commonly observed in dogs with HAC [9,10]. However, studies of cardiac disease in dogs with HAC are relatively limited. Hypertrophic cardiomyopathy or LVH is not commonly observed in dogs and accounts for only 5% of canine cardiac disease [11]. The aims of the study were to report the prevalence of LVH and to assess the left ventricular systolic and diastolic function using two-dimensional speckle-tracking echocardiography (2D-STE) in small breed dogs with HAC.

## Methods

### Animals

#### Age-matched healthy controls (controls)

Client-owned dogs visiting the National Taiwan University Veterinary Hospital (NTUVH) during 2010 and 2012 for routine wellness checkups were included in this study. Thorough medical histories were obtained, and physical examinations, routine blood analyses (complete blood counts and biochemical profiles), chest radiographic examinations, abdominal ultrasonographic examinations, echocardiographic examinations (two-dimensional (2D), M-mode, and Doppler) and 2D-STE were performed to exclude the presence of any forms of systemic, cardiac, renal, and adrenal disease. Nine dogs (five males and four females) were deemed clinically healthy and categorized as age-matched healthy controls (controls).

#### Dogs with hyperadrenocorticism (HAC group)

Client-owned dogs affected with HAC during the same period of time were included in this group. Inclusion criteria of HAC included evidence of clinical signs consistent with a diagnosis of HAC (e.g., polydipsia, polyuria, polyphagia, decreased activity, panting, a potbellied appearance, and dermatologic problems), results of routine serum biochemical analyses consistent with a diagnosis of HAC (i.e., elevated activities of hepatic enzymes), and results of ACTH stimulation tests consistent with a diagnosis of HAC. Dogs with inconclusive results from ACTH stimulation tests were excluded from the study. All included dogs with HAC were required to have received an ACTH stimulation test with intramuscular injection of 0.25 mg of synthetic ACTH (Cortrosyn; Organon, the Netherlands) prior to treatment. Blood samples for the detection of serum cortisol concentrations were collected via cephalic venipuncture immediately before and one hour after synthetic ACTH was injected [12]. Serum cortisol concentrations were measured by use of a validated radioimmunoassay (Coat-A-Count Cortisol; Diagnostic Products Corp., Los Angeles, California). Further differentiation between PDH and ADH was based upon adrenal ultrasonographic assessment [13,14]. Normal or mildly and bilaterally enlarged adrenal glands were classified as PDH, whereas ultrasonographic findings of adrenals with asymmetry, abnormal enlargement, or irregular or round shape, or nodules/masses identified in the entire adrenal or in one pole - with heterogenicity and mineralized foci - were diagnosed as ADH [13,14]. Equivocal cases and cases that presented with bilaterally and irregularly enlarged/round adrenals were excluded from the study.

### Systolic blood pressure measurement

Systolic blood pressure was measured using the indirect method of Doppler flow detection with a 9.5 MHz transducer. A Doppler Ultrasonic Flow Detector (Model 811-B, Parks Medical Electronics Inc., U.S.A.) with an inflatable cuff width of 1.9 or 2.5 cm (approximately 40% of the circumference of the antebrachium) was used. During systemic blood pressure measurement, the antebrachium was maintained at the level of the heart. A series of five readings (with 10 to 20 seconds between consecutive measurements) was obtained for each dog. To minimize procedural stress, all dogs were allowed to assume a comfortable position with only gentle restraint by their owners. The dogs remained in the same position throughout systemic blood pressure measurement. The final systemic blood pressure value was calculated as the mean of five readings. The heart rate was manually recorded by pulse Doppler ultrasound detection over a period of 20 seconds after the systemic blood pressure value was measured. Dogs were considered to be hypertensive if the average systemic blood pressure exceeded 160 mm Hg [15].

### Conventional echocardiography

Conventional echocardiography was performed without sedation in gently restrained recumbent positions. Standard echocardiographic views were obtained in right and left lateral recumbency using the 2D-guided M mode with ultrasound units equipped with 2–5 and 5–7.5 MHz transducers (MyLab™ 50 Family, Esaote, Genova, Italy). Measurements of left ventricular end-diastolic diameter (LVEDD), left ventricular end-systolic diameter (LVESD), left ventricular free-wall thickness (LVFWd) and interventricular septal thickness (IVSd) at end-diastole, ejection fraction (EF), and fractional shortening (FS) were obtained from the right parasternal long-axis view. The left atrium to aorta ratio (LA/AO) was measured in the right parasternal short-axis view. Values for the inflow of the maximal early (E) and late (A) diastolic mitral and tricuspid flow velocities, E/A ratio, and isovolumic relaxation time (IVRT) were determined by pulsed-wave Doppler from the left apical four- or five-chamber view.

### Inclusion criteria of left ventricular hypertrophy

Inclusion of LVH was based on conventional echocardiographic examinations. LVH was defined as regional or generalized concentric hypertrophy with a diastolic wall thickness ≧ 7 mm of the LVFWd or ≧ 6 mm of the IVSd in dogs with a body weight of < 5 kg, and with a diastolic wall thickness of ≧ 8 mm of the LVFWd or ≧ 7 mm of the IVSd in dogs with a body weight of 5–11 kg [16].

### Two-dimensional speckle-tracking echocardiography

**Figure 1:**
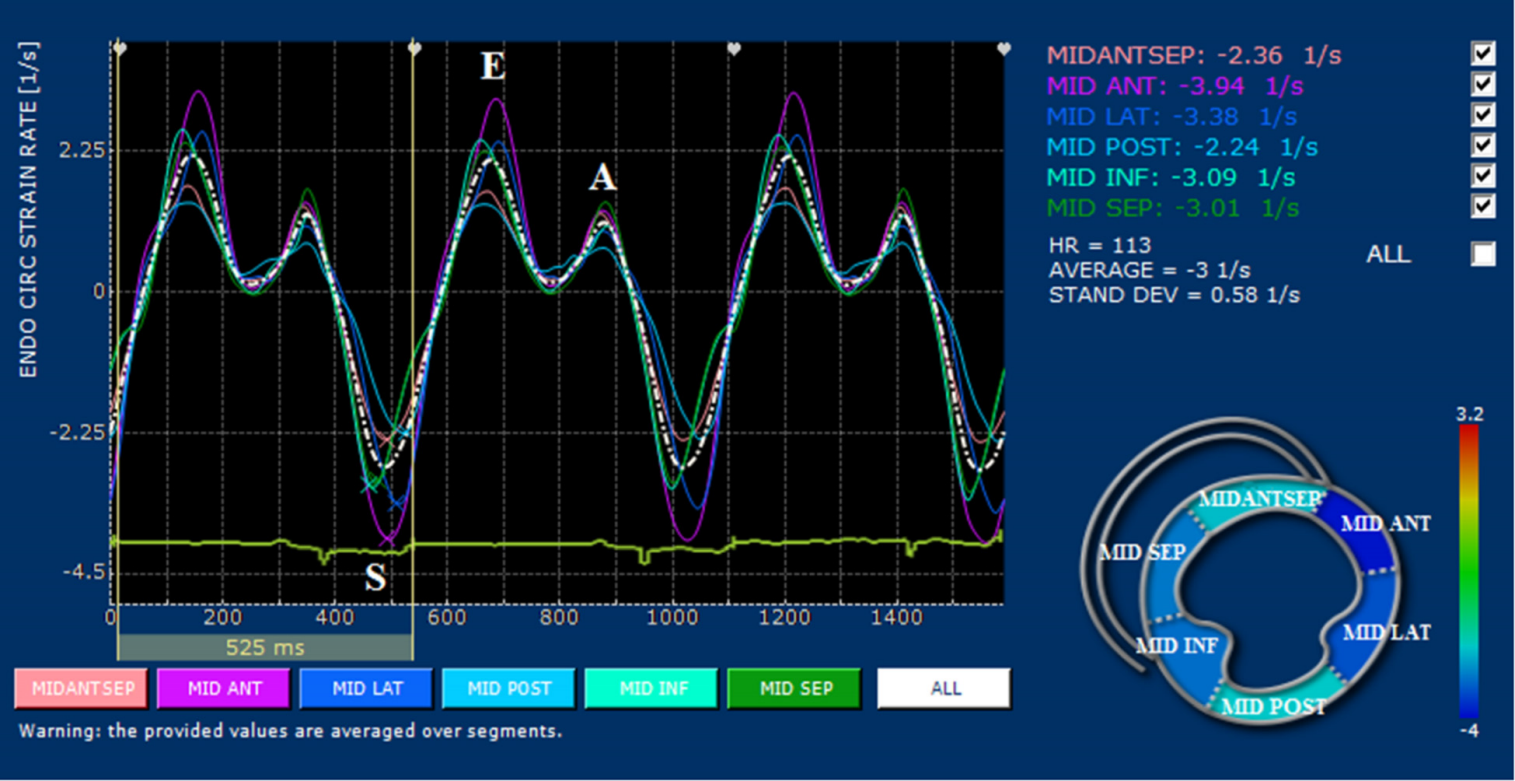
**Two-dimensional speckle-tracking echocardiography analysis of left ventricular circumferential strain in a dog (15 years old male Maltese terrier, 4.0 kg) with pituitary-dependent hyperadrenocorticism.** The left ventricular myocardium is automatically divided into six segments in the short axis view: middle anterior septal wall (MIDANTSEP), middle anterior left ventricular wall (MID ANT), middle lateral left ventricular wall (MID LAT), middle posterior left ventricular wall (MID POST), middle inferior left ventricular wall (MID INF), middle ventricular septum (MID SEP).

**Figure 2:**
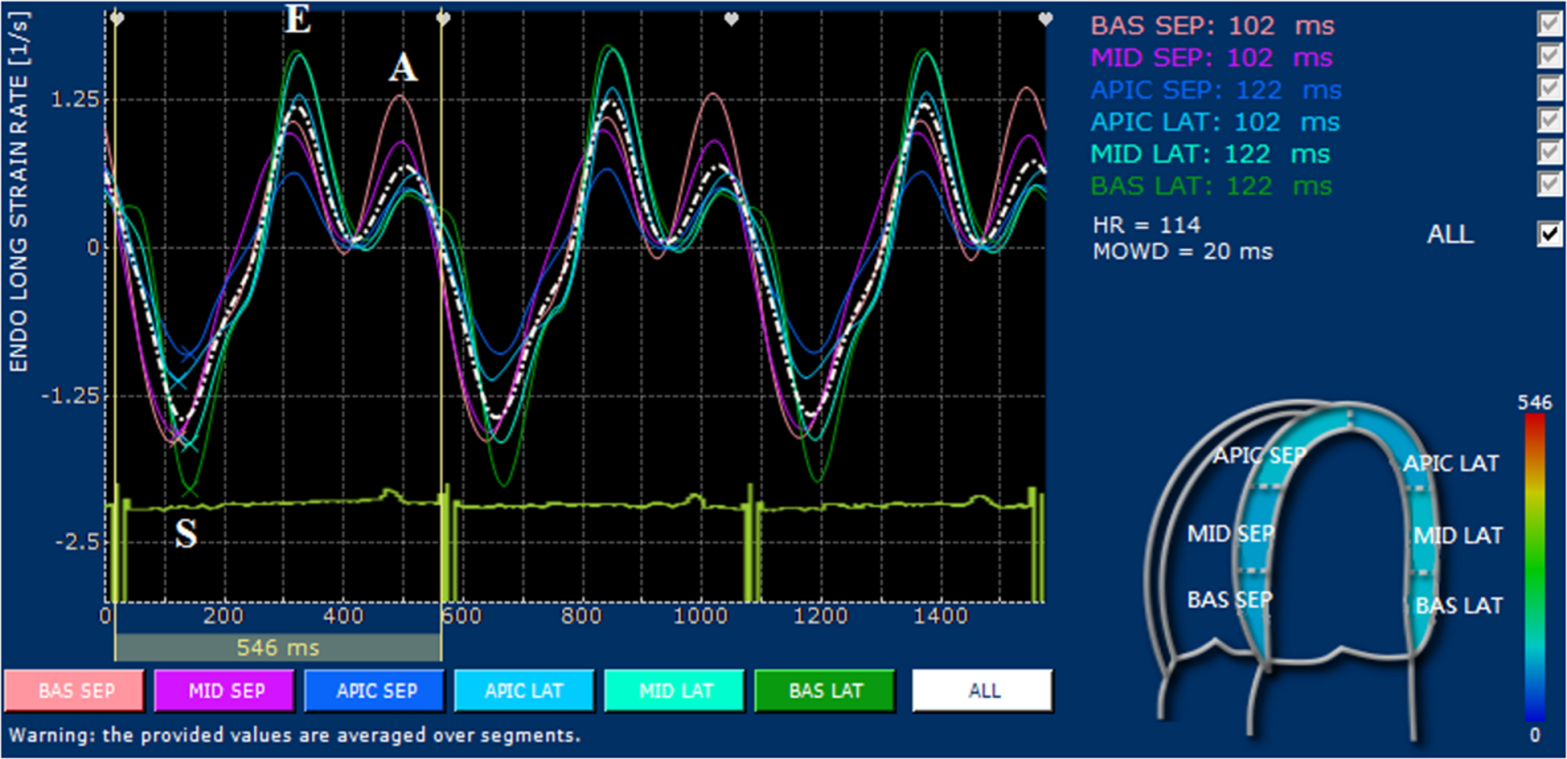
**Two-dimensional speckle-tracking echocardiography analysis of left ventricular longitudinal strain in a dog (10 years old male Maltese terrier, 3.2 kg) with pituitary-dependent hyperadrenocorticism.** The left ventricular myocardium is automatically divided into six segments: basal (BAS SEP), middle (MID SEP) and apical (APIC SEP) segments for the interventricular septum, and basal (BAS LAT), middle (MID LAT) and apical (APIC LAT) lateral segments for the left ventricular free wall.

2D-STE examinations were performed at the end of conventional echocardiography for all dogs. The standard left apical long-axis four-chamber and the right parasternal short-axis views at the level of papillary muscles were obtained using a frame rate above 60 per second. For each dog, three consecutive cardiac cycles were recorded using one single loop, and digitally stored in a hard disk for offline analysis on a workstation (XStrain™ software for MyLab™ 50 XVision, Esaote, Genova, Italy). The 2D-STE variables included strain, and strain rate. Circumferential strain rate in peak systole (CSR_S_), circumferential strain rate in early diastole (CSR_E_), circumferential strain rate in late diastole (CSR_A_) and peak circumferential strain (CS) were measured from the right parasternal short-axis view at the level of the papillary muscles. The software automatically divided the left ventricle into six segments based on standard segmentation model used in human patients [17] (Figure 1). Longitudinal strain rate in peak systole (LSR_S_), longitudinal strain rate in early diastole (LSR_E_), longitudinal strain rate in late diastole (LSR_A_), and peak longitudinal strain (LS) were measured from the left apical long-axis four-chamber view. The software automatically divided into three segments in each the left ventricular free wall and interventricular septum based on standard segmentation model used in human patients [18] (Figure 2). Only images with > 4/6 segments of adequate tracking quality were included for analysis. Global strain and strain rate were calculated by averaging the values of all segmental circumferential/longitudinal strain and strain rate.

### Statistical analysis

Data are presented as mean ± standard deviation (SD). Descriptive statistical analyses were performed with a with commercial computer statistics software (Statistical Product and Service Solutions (SPSS) Version 12.0.1, SPSS Inc. in Chicago, IL, USA.). Continuous data were tested for normality by use of the D’Agostino-Pearson omnibus test and Shapiro-Wilk test. All tests were two-tailed, and a *P* value < 0.05 was considered statistically significant. Normal distribution was found in all continuous data including, variables derived from conventional echocardiography and STE.

Overall group-wise in continuous data (age, heart rate, systemic blood pressure, body weight), as well as conventional echocardiographic and STE-derived variables, among the PDH, ADH, and control groups were investigated using analysis of variance and post hoc tests with a Scheffe-adjusted *P* value for multiple comparisons. The relationships between STE-derived variables and age, systemic blood pressure, body weight, LVFWd, and IVSd were assessed using Pearson’s correlation coefficient.

Least squares multiple linear regression analyses were performed with the measured STE-derived variables as dependent variables and the independent variables of gender, age, heart rate, systemic blood pressure, IVSd, and LVFWd. A Chi-square test was used to assess differences in categorical variables among the groups. Comparison of the systemic blood pressure, conventional echocardiographic variables, and 2D-STE derived variables between LVH dogs and controls was performed using Student’s unpaired two-tailed *t* tests.

The intra-observer coefficients of variations (CV) of the conventional echocardiographic and 2D-STE derived variables were calculated by a variance component analysis. The CV was determined by dividing the SD value by the overall mean. Six clinically healthy dogs were scanned on three nonconsecutive occasions within one day by the same examiner (HYC), who was unaware of values from the initial examination.

## Results

### Baseline descriptions of the animals

The breeds represented in the controls included one miniature dachshund and two each of Maltese terriers, miniature Schnauzers, mixed breeds, and Yorkshire terriers. The mean age of the control was 10.5 ± 2.8 years (range: 7 to 15 years) and the mean body weight was 6.2 ± 2.7 kg (range: 2.6 to 10.0 kg). Nineteen dogs with HAC were included: 10 dogs with pituitary-dependent hyperadrenocorticism (PDH) and nine dogs with adrenal-dependent hyperadrenocorticism (ADH). The breed distribution of the 19 dogs with HAC was as follows: eight Maltese terriers, five Shih-Tzus, and one each of Chihuahua, miniature Schnauzer, Pomeranian, toy poodle, short-haired dachshund, and Yorkshire terrier. The mean age of the 10 dogs (five males and five females) with PDH was 12.5 ± 2.0 years (range: 9 to 15 years) and the mean body weight of this groups was 4.5 ± 1.4 kg (range: 3.2 to 6.6 kg). The mean age of the nine dogs (three males and six females) with ADH was 12.3 ± 2.0 years (range: 9 to 15 years), and the mean body weight of this group was 5.9 ± 2.7 kg (range: 2.1 to 10.0 kg).

No statistically significant differences in body weight and heart rate among controls, the PDH, and the ADH groups were found (Table 1). The female to male ratio in controls, the PDH group, and the ADH group was 0.8, 1.0, and 2.0, respectively. The gender distribution was not significantly different among groups (*P* = 0.615).

**Table 1:** **Baseline characteristics in the control, pituitary-dependent hyperadrenocorticism (PDH), and adrenal-dependent hyperadrenocorticism (ADH) groups**

**Variables**	**Control (** ***n*** **= 9)**	**PDH (** ***n*** **= 10)**	**ADH (** ***n*** **= 9)**
**Age (y/o)**	10.5 ± 2.8	12.5 ± 2.0	12.3 ± 2.0
**Body weight (kg)**	6.2 ± 2.7	4.5 ± 1.4	5.9 ± 2.7
**Female to male**	0.8	1	2
**Heart rate (bpm)**	128.9 ± 23.4	124.6 ± 16.8	129.3 ± 13.7
**Blood pressure (mmHg)**	134.6 ± 11.1	140.4 ± 11.0	156.2 ± 16.5^‡†^
**Incidence of hypertension (%)**	0%	10%	56%^‡†^
**LVEDD (mm)**	24.9 ± 3.8	23.6 ± 7.0	21.0 ± 4.8
**LVESD (mm)**	14.1 ± 2.8	12.9 ± 3.8	12.3 ± 3.1
**LVFWd (mm)**	4.9 ± 1.1	6.3 ± 1.0^†^	6.4 ± 1.0^†^
**IVSd (mm)**	5.1 ± 0.8	6.6 ± 1.2^†^	7.1 ± 1.0^‡†^
**Incidence of LVH (%)**	0%	40%^†^	56%^†^
**LA/Ao ratio**	1.4 ± 0.1	1.5 ± 0.2	1.4 ± 0.2
**EF (%)**	75.4 ± 9.5	79.5 ± 5.6	75.0 ± 6.1
**FS (%)**	43.0 ± 8.0	46.1 ± 5.3	41.3 ± 5.1
**Mitral peak E wave (m/s)**	0.82 ± 0.33	0.63 ± 0.13	0.52 ± 0.20^†^
**Mitral E/A ratio**	1.07 ± 0.35	0.94 ± 0.29	0.75 ± 0.38
**Tricuspid peak E wave (m/s)**	0.50 ± 0.16	0.41 ± 0.10	0.46 ± 0.23
**Tricuspid E/A ratio**	0.97 ± 0.31	0.94 ± 0.23	0.86 ± 0.43
**IVRT (mm)**	47.9 ± 8.7	57.4 ± 13.7	64.2 ± 20.3^‡†^
**E/IVRT**	1.51 ± 0.34	0.92 ± 0.30^†^	0.89 ± 0.42^†^

Indices are presented as mean ± SD.

E/A ratio, early diastolic velocity/late diastolic velocity ratio; EF, ejection fraction; FS, fractional shortening; IVRT, isovolumetric relaxation time; IVSd, thickness of interventricular septum at end-diastole; LA/Ao, left atrial/aortic root ratio; LVEDD, left ventricular dimension at end-diastole; LVESD, left ventricular dimension at end-systole; LVFWd, thickness of left ventricular free wall at end-diastole, LVH, left ventricular hypertrophy.

^†^ *P* < 0.05 relative to controls; ^‡^ *P* < 0.05 relative to PDH.

### Systemic blood pressure and conventional echocardiography

The mean systemic blood pressure was highest in the ADH group (*P* = 0.005 versus PDH group and controls, Table 1). Prevalence of hypertension was also highest in the ADH group (*P* = 0.009 versus PDH group and controls, Table 1).

In conventional echocardiographic examinations, most indices among controls, the PDH, and the ADH groups were not different, except LVFWd and IVSd, mitral E wave, and IVRT values (Table 1). Both LVFWd and IVSd were greatest in the ADH group, followed by the PDH group and controls (*P* = 0.014 and *P* = 0.001, respectively, Table 1). LVH was diagnosed in 56% (5/9) of dogs with ADH and 40% (4/10) of dogs with PDH (*P* = 0.656). Five dogs (ADH, *n* = 3; PDH, *n* = 2) were characterized with symmetric LVH, four dogs (ADH, *n* = 2; PDH, *n* = 2) were characterized with focal and asymmetric LVH.

IVRT was highest in ADH, followed by PDH and controls (*P* = 0.008, Table 1). Mitral E wave and E/IVRT was significantly higher in controls, followed by PDH and ADH (*P* = 0.036 and *P* = 0.001, respectively, Table 1). Neither LVFWd nor IVSd was correlated with systemic blood pressure (*r*^2^ = 0.05, *P* = 0.238 and *r*^2^ = 0.09, *P* = 0.113, respectively). Neither LVFWd nor IVSd was correlated with body weight (*r*^*2*^ = 0.05, *P* = 0.238 and *r*^2^ = 0.02, *P* = 0.482, respectively). Systemic blood pressure was negatively correlated with E/IVRT (*r*^2^ = −0.19, *P* = 0.019).

No significant differences in systemic blood pressure, FS, or EF were found between dogs with LVH (*n* = 9) and controls (*n* = 10; *P* = 0.125, *P* = 0.625, and *P* = 0.843, respectively). No significant differences in systemic blood pressure was found between HAC dogs with LVH (*n* = 9) and without LVH (*n* = 10; *P* = 0.935). Significant decreases in mitral E/A ratio and significant increases in IVRT were also found in LVH dogs compared to controls (*P* = 0.041 and *P* = 0.032, respectively).

### Left ventricular strain and strain rate

**Figure 3:**
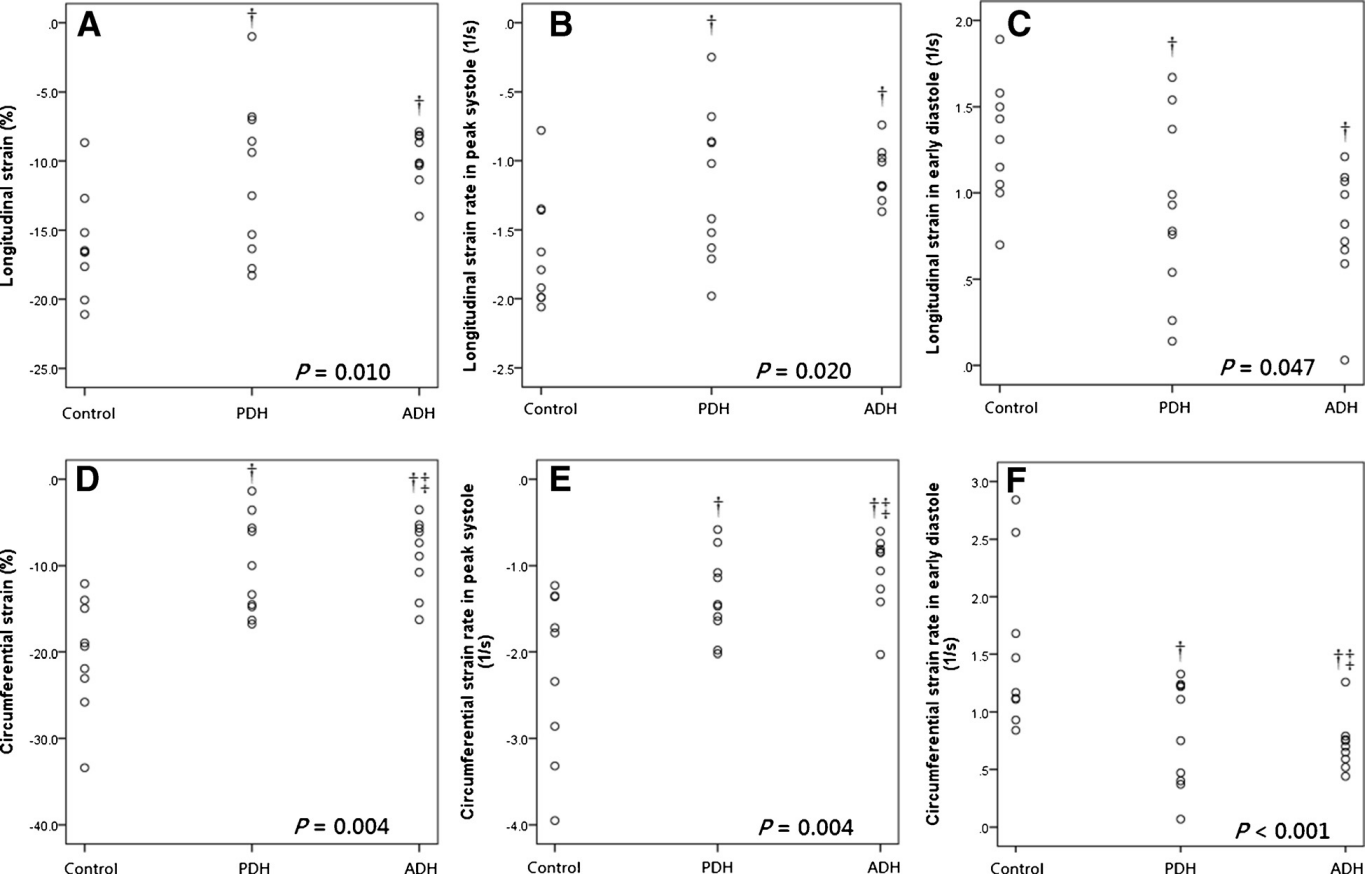
**Dot-plots of (A) global peak longitudinal strain, (B) global longitudinal strain rate in peak systole, (C) global circumferential strain in early diastole, (D) global peak circumferential strain, (E) global circumferential strain rate in peak systole and (F) circumferential strain rate in early diastole derived from two-dimensional speckle-tracking echocardiography in age-matched healthy controls and dogs affected with pituitary-dependent hyperadrenocorticism (PDH) and adrenal-dependent hyperadrenocorticism (ADH).** ^†^ *P* < 0.05 relative to controls; ^‡^ *P* < 0.05 relative to PDH.

Almost all variables derived from the segmental and global strain/strain rate in longitudinal and circumferential dynamics followed the same pattern: highest in the controls, followed by the PDH group, then the ADH group (Tables 2, 3, 4 and 5). Seven of eight global 2D-STE variables, namely global LSR_S_, LSR_E_, LSR_A_, LS, CSR_S_, CSR_E_, and CS, were significantly different among the ADH, PDH, and control groups (*P* = 0.020, *P* = 0.047, *P* = 0.049, *P* = 0.010, *P* = 0.004, *P* = 0.004 and *P* < 0.001, respectively, Table 2, Figure 3). Results of the segmental STE derived from control and the HAC groups are provided in Tables 3, 4 and 5.

**Table 2:** **Global strains and strain rates derived from two-dimensional speckle-tracking echocardiography in the control, pituitary-dependent hyperadrenocorticism (PDH), and adrenal-dependent hyperadrenocorticism (ADH) groups**

**Variables**	**Control (** ***n*** **= 9)**	**PDH (** ***n*** **= 10)**	**ADH (** ***n*** **= 9)**
**Global LSR**_**S**_ **(s** ^**−1**^ **)**	−1.66 ± 0.42	−1.19 ± 0.54^†^	−1.11 ± 0.20^†^
**Global LSR**_**E**_ **(s** ^**−1**^ **)**	1.29 ± 0.36	0.90 ± 0.51^†^	0.80 ± 0.36^†^
**Global LSR**_**A**_ **(s** ^**−1**^ **)**	0.95 ± 0.41	0.54 ± 0.25^†^	0.62 ± 0.40^†^
**Global CSR**_**S**_ **(s** ^**−1**^ **)**	−2.21 ± 0.97	−1.37 ± 0.48^†^	−1.07 ± 0.44^‡†^
**Global CSR**_**E**_ **(s** ^**−1**^ **)**	1.52 ± 0.71	0.82 ± 0.46^†^	0.72 ± 0.23^‡†^
**Global CSR**_**A**_ **(s** ^**−1**^ **)**	0.82 ± 0.35	0.73 ± 0.42	0.60 ± 0.30
**Global LS (%)**	−16.11 ± 3.73	−11.29 ± 5.67^†^	−9.87 ± 1.97^†^
**Global CS (%)**	−20.39 ± 6.61	−10.22 ± 5.67^†^	−8.68 ± 4.32^‡†^

A: late diastole; CS: circumferential strain; CSR: circumferential strain rate; E: early diastole; LS: longitudinal strain; LSR: longitudinal strain rate; S: peak systole.

^†^ *P* < 0.05 relative to controls; ^‡^ *P* < 0.05 relative to PDH.

**Table 3:** **Indices of left ventricle longitudinal strain rates (LRS) derived from two-dimensional speckle-tracking echocardiography in the control, pituitary-dependent hyperadrenocorticism (PDH), and adrenal-dependent hyperadrenocorticism (ADH) groups**

**Variables**		**LSR**_**S**_ **(s** ^**−1**^ **)**	**LSR**_**E**_ **(s** ^**−1**^ **)**	**LSR**_**A**_ **(s** ^**−1**^ **)**
**Basal septal**	Control	−1.64 ± 0.43	1.44 ± 0.40	0.91 ± 0.37
	PDH	−1.29 ± 0.57	1.05 ± 0.50	0.67 ± 0.32
	ADH	−1.38 ± 0.37	1.05 ± 0.27	0.90 ± 0.61
**Mid septal**	Control	−1.72 ± 0.34	1.08 ± 0.42	1.01 ± 0.26
	PDH	−1.39 ± 0.72	0.97 ± 0.56	0.78 ± 0.26
	ADH	−1.40 ± 0.20	0.98 ± 0.41	0.83 ± 0.61
**Apical septal**	Control	−1.66 ± 0.82	0.90 ± 0.54	0.98 ± 0.46
	PDH	−1.53 ± 1.04	1.14 ± 0.83	0.81 ± 0.51
	ADH	−1.39 ± 0.61	0.91 ± 0.33	0.81 ± 0.86
**Apical lateral**	Control	−1.56 ± 0.77	1.07 ± 0.58	0.75 ± 0.47
	PDH	−1.25 ± 0.66	1.02 ± 0.71	0.65 ± 0.42
	ADH	−1.00 ± 0.57	0.70 ± 0.35	0.55 ± 0.52
**Mid lateral**	Control	−1.69 ± 0.76	1.45 ± 0.76	0.75 ± 0.40
	PDH	−1.22 ± 0.60	1.04 ± 0.50	0.59 ± 0.36
	ADH	−0.97 ± 0.38^†^	0.75 ± 0.49	0.59 ± 0.29
**Basal lateral**	Control	−1.87 ± 1.01	1.71 ± 1.14	0.84 ± 0.55
	PDH	−1.52 ± 0.80^†^	1.17 ± 0.60	0.72 ± 0.37
	ADH	−1.13 ± 0.28^‡†^	1.04 ± 0.66	0.68 ± 0.32

A: late diastole; E: early diastole; S: peak systole.

^†^ *P* < 0.05 relative to controls; ^‡^ *P* < 0.05 relative to PDH.

**Table 4:** **Indices of left ventricle circumferential strain rates (CRS) derived from two-dimensional speckle-tracking echocardiography in the age-matched control, pituitary-dependent hyperadrenocorticism (PDH), and adrenal-dependent hyperadrenocorticism (ADH) groups**

**Variables**		**CSR**_**S**_ **(s** ^**−1**^ **)**	**CSR**_**E**_ **(s** ^**−1**^ **)**	**CSR**_**A**_ **(s** ^**−1**^ **)**
**Mid antsep**	Control	−2.04 ± 0.48	1.37 ± 0.54	0.78 ± 0.27
	PDH	−1.01 ± 0.50^†^	0.63 ± 0.48^†^	0.71 ± 0.49
	ADH	−1.35 ± 0.86^†^	0.91 ± 0.41^‡†^	0.67 ± 0.36
**Mid ant**	Control	−2.09 ± 0.78	1.28 ± 0.49	0.83 ± 0.25
	PDH	−1.31 ± 0.54	0.64 ± 0.34^†^	0.88 ± 0.52
	ADH	−1.58 ± 0.89	0.92 ± 0.55^‡†^	0.93 ± 0.50
**Mid lat**	Control	−2.26 ± 0.78	1.61 ± 0.62	0.83 ± 0.30
	PDH	−1.52 ± 0.67	1.21 ± 0.52	0.85 ± 0.56
	ADH	−1.43 ± 0.57^†^	0.88 ± 0.37^†^	0.89 ± 0.46
**Mid post**	Control	−2.58 ± 0.83	1.83 ± 0.65	1.03 ± 0.58
	PDH	−1.67 ± 0.66^†^	1.48 ± 0.78	0.78 ± 0.67
	ADH	−1.25 ± 0.57^‡†^	0.89 ± 0.45^†^	0.75 ± 0.43
**Mid inf**	Control	−2.39 ± 0.88	1.85 ± 0.52	1.02 ± 0.46
	PDH	−1.70 ± 0.68	1.06 ± 0.42	0.92 ± 0.49
	ADH	−1.01 ± 0.49^†^	0.75 ± 0.45^†^	0.59 ± 0.38
**Mid sep**	Control	−2.06 ± 0.56	1.59 ± 0.52	0.77 ± 0.27
	PDH	−1.34 ± 0.72^†^	0.90 ± 0.42^†^	0.78 ± 0.50
	ADH	−1.05 ± 0.52^‡†^	0.92 ± 0.27^‡†^	0.48 ± 0.37

A: late diastole; Ant: anterior left ventricular wall; Antsep: anterior septal wall; E: early diastole; Inf: inferior left ventricular wall; Lat: lateral left ventricular wall; Mid: middle; Post: posterior left ventricular wall; S: peak systole; Sep: ventricular septum.

^†^ *P* < 0.05 relative to controls; ^‡^ *P* < 0.05 relative to PDH.

**Table 5:** **Indices of longitudinal (LS) and circumferential strains (CS) of left ventricle derived from two-dimensional speckle-tracking echocardiography in the age-matched control, pituitary-dependent hyperadrenocorticism (PDH), and adrenal-dependent hyperadrenocorticism (ADH) groups**

**Variables**	**Peak LS (%)**	**Variables**	**Peak CS (%)**
**Basal septal**		**Mid antsep**	
Control	−16.79 ± 3.97	Control	−17.87 ± 4.27
PDH	−12.80 ± 5.67	PDH	−7.86 ± 4.66^†^
ADH	−11.44 ± 3.60	ADH	−10.16 ± 6.92^‡†^
**Mid septal**		**Mid ant**	
Control	−16.99 ± 4.45	Control	−18.31 ± 4.06
PDH	−13.73 ± 7.21	PDH	−9.38 ± 5.90^†^
ADH	−12.58 ± 1.71	ADH	−11.66 ± 8.16^‡†^
**Apical septal**		**Mid lat**	
Control	−15.13 ± 6.64	Control	20.21 ± 5.94
PDH	−14.89 ± 10.34	PDH	10.51 ± 6.44^†^
ADH	−12.27 ± 4.68	ADH	10.24 ± 5.44^‡†^
**Apical lateral**		**Mid post**	
Control	−14.51 ± 5.47	Control	21.48 ± 7.48
PDH	−12.64 ± 8.53	PDH	12.61 ± 7.07^†^
ADH	−8.52 ± 4.85	ADH	8.80 ± 3.95^‡†^
**Mid lateral**		**Mid inf**	
Control	−16.32 ± 9.30	Control	21.16 ± 7.20
PDH	−9.80 ± 5.51	PDH	13.03 ± 6.66^†^
ADH	−8.19 ± 4.53^†^	ADH	7.44 ± 3.82^‡†^
**Basal lateral**		**Mid sep**	
Control	−18.46 ± 3.93	Control	18.23 ± 5.25
PDH	−10.51 ± 6.16^†^	PDH	10.16 ± 5.42^†^
ADH	−9.84 ± 4.64^‡†^	ADH	7.56 ± 3.58^‡†^

A: late diastole; Ant: anterior left ventricular wall; Antsep: anterior septal wall; E: early diastole; Inf: inferior left ventricular wall; Lat: lateral left ventricular wall; Mid: middle; Post: posterior left ventricular wall; S: peak systole; Sep: ventricular septum.

^†^ *P* < 0.05 relative to controls; ^‡^ *P* < 0.05 relative to PDH.

Systemic blood pressure was negatively correlated with global CRS_E_ (*r*^2^ = −0.16, *P* = 0.036). No correlation between age, body weight, and 2D-STE indices were found.

The IVSd was negatively correlated with global LSR_S_ (*r*^2^ = −0.16, *P* = 0.033), global LSR_E_ (*r*^2^ = −0.14, *P* = 0.048), global LS (*r*^2^ = −0.28, *P* = 0.004), global CSR_S_ (*r*^*2*^ = −0.15, *P* = 0.004) and global CS (*r*^2^ = −0.25, *P* = 0.007). The LVFWd was negatively correlated with global LSR_S_ (*r*^2^ = −0.15, *P* = 0.043), global LS (*r*^*2*^ = −0.25, *P* = 0.007) and global CS (*r*^2^ = −0.16, *P* = 0.031). The E/IVRT was positively correlated with global CSR_S_ (*r*^*2*^ = −0.27, *P* = 0.005), global CSR_E_ (*r*^2^ = −0.32, *P* = 0.002) and global CS (*r*^2^ = −0.29, *P* = 0.002).

Seven of eight global 2D-STE variables, namely LSR_S_, LSR_E_, LS, global CSR_S_, CSR_E,_ and CS, were significantly decreased in dogs with LVH compared to controls (*P* = 0.004, *P* = 0.009, *P* = 0.002, *P* = 0.014, *P* = 0.021 and *P* = 0.001, respectively); however, these differences between dogs with LVH (*n* = 9) and without LVH (*n* = 10) in the HAC group did not reach statistical significance.

### Multiple regression analyses

In the multiple regression analyses, global LS, global LSR_S_, and LSR_E_ all decreased linearly with IVSd and were affected by gender (adjusted *r*^2^ = 0.531, *P* < 0.003; adjusted *r*^2^ = 0.408, *P* = 0.001 and adjusted *r*^2^ = 0.214, *P* = 0.019, respectively), IVSd had the highest *r*^2^ in the final model.

Global CS decreased linearly with IVSd (adjusted *r*^2^ = 0.223, *P* = 0.007). Global CSR_S_ decreased linearly with IVSd and increased linearly with heart rate (adjusted *r*^2^ = 0.270, *P* = 0.008), IVSd had the highest *r*^2^ in the final model. Global CSR_E_ increased linearly with heart rate and decreased linearly with systemic blood pressure (adjusted *r*^2^ = 0.328, *P* = 0.003), systemic blood pressure had the highest *r*^2^ in the final model.

### The intra-observer coefficients of variations (CV) of the echocardiographic examinations

CV (%) of 2D-STE variables were all less than 20% (Table 6).

**Table 6:** **Within-day intra-observer variability of global strain (%) and strain rate (1/second) in longitudinal and circumferential dynamics from six dogs**

	**Within-Day CV%**	**Within-Day SD**
**LS (%)**	13.3	1.25
**LSR**_**S**_ **(1/s)**	10.6	0.12
**LSR**_**E**_ **(1/s)**	11.6	0.10
**LSR**_**A**_ **(1/s)**	16.3	0.09
**CS (%)**	6.7	1.25
**CSR**_**S**_ **(1/s)**	11.7	0.20
**CSR**_**E**_ **(1/s)**	14.3	0.22
**CSR**_**A**_ **(1/s)**	15.1	0.09

A: late diastole; CS: circumferential strain; CSR: circumferential strain rate; CV, coefficient of variation; E: early diastole; LS: longitudinal strain; LSR: longitudinal strain rate; S: peak systole; SD: standard deviation.

## Discussion

Chronic excessive cortisol concentrations might cause hypertension. Subsequent left ventricular remodeling, hypertrophy, and altered diastolic function are the most important risk factors of cardiovascular mortality in human patients with HAC [19]. Sixty-eight to eighty-five per cents of human HAC patients developed hypertension [20,21]. Likewise, systemic hypertension occurs in 47 to 86% of HAC cases in dogs [9,22-24]. In this study, the prevalence of hypertension in dogs with HAC was 32%. This prevalence of hypertension was low compared to the other studies, but was consistent with the result reported in our previous study [10] and was significantly higher than that of age-matched control dogs. This finding supported that dogs with HAC may be prone to developing hypertension. In this study, the condition of HAC was recently diagnosed. Systemic blood pressure was measured at the time of the initial diagnosis. A higher prevalence of hypertension may be observed at later stages of the condition.

Cardiac hypertrophy secondary to hypertension and chronic exposure to excess circulating cortisol may also contribute directly to left ventricular concentric remodeling in human patients with HAC. Subsequently, functional abnormality, such as diastolic dysfunction (reduced E/A ratio and/or E velocity) has also been reported in human patients with HAC [25,26]. In this study, the left ventricular wall thickness, IVSd, and LVFWd were significantly increased in the ADH and PDH groups compared with the age-matched control group. Significant decreases in mitral E wave and increases in IVRT were also found in the ADH and PDH groups compared to the controls. These findings indicated that dogs with HAC are prone to present with LVH, impaired LV relaxation and diastolic dysfunction. In our study, LVH was diagnosed in 47.3% of dogs with HAC. Based upon the result of this study, LVH was more prevalent in small breed dogs with HAC compared to controls. Although chronic degenerative valvular disease is the most common acquired cardiac disease in small breeds of dogs [27], LVH should be a differential diagnosis in small breed dogs with HAC.

Development of LVH is generally believed to be associated with concomitant hypertension in HAC. However in this study, the wall thickness of the left ventricle was not correlated with systemic blood pressure. Significant differences in systemic blood pressure were not found between dogs with LVH and controls, or between HAC dogs with LVH and HAC dogs without LVH. No association between hypertension and development of LVH was identified in this study. The mechanism of development of LVH in dogs with HAC could not be clarified in this study.

In 2D-STE analysis, global peak systolic and early diastolic strain rate, as well as global strain in longitudinal and circumferential dynamics exhibited a consistent pattern: profound decreases in the ADH and the PDH groups compared to controls, i.e. significant decreases in systolic and diastolic functions were observed in the ADH and PDH groups when compared to controls. Left ventricular systolic and diastolic dysfunction was also reported in human patients with HAC [5,28]. Diastolic dysfunction is associated with LVH, whereas systolic dysfunction is associated with LVH and myocardial fibrosis [5,28]. Upon remission of HAC, left ventricular parameters ameliorate considerably, but does not fully restored after remission [4].

In this study, 2D-STE revealed alterations in left ventricular mechanics despite a preserved systolic function detected by conventional echocardiography in dogs with HAC. The indices of left ventricular systolic function derived from conventional echocardiography, such as FS, EF, LVEDD and LVESD, were not significantly different between the HAC group and controls. FS and EF did not reach significant difference between dogs with LVH and the control. However decreased systolic function was revealed using 2D-STE. Decreased systolic function was most prominent in dogs with LVH associated with ADH, with decreasing prominence observed in the PDH group and finally in controls. This finding suggests that dogs with HAC may have subclinical systolic dysfunction that may remain undetected using conventional echocardiographic examination. Similar findings are also reported in human patients with HAC-associated hypertrophic cardiomyopathy: subclinical left ventricular systolic dysfunction is indicated using STE, although the assessment of FS and EF is usually normal or supernormal [25,29].

Global LS, global LSR_S_, global LSR_E_, global CS, and CSR_S_ decreased while IVSd increased, suggesting that decreased magnitude and rate of myocardial deformation is followed by increased LVH. Based on these findings, increased IVSd could be used as predictor of subclinical LV dysfunction in small breed dogs with HAC. In addition, global CSR_E_ decreased linearly with increased systemic blood pressure. Based on this finding, increased systemic blood pressure could be used as predictor of subclinical LV diastolic dysfunction in small breed dogs with HAC.

E/IVRT is an index to predict for left ventricular filling pressure, and increases when LV filling pressure is increased in dogs with congestive heart failure characterized by volume overload [30]. In this study, decreased E/IVRT was found in dogs with HAC. This finding suggested that dogs with HAC might have decreased left ventricular filling pressure compared to the controls. This phenomenon might be caused by the decreased volume load of body fluid associated with the diuretic effect of elevated cortisol concentration in dogs with HAC.

Longitudinal deformation was affected by gender in this study. Although dogs with HAC are more commonly female, no gender predilection has been reported. In this study, 57% of the dogs with HAC were females, a rate similar to that previously reported [10]; however, the effect of gender on STE has not been previously reported and the precise effect of gender on longitudinal deformation has been unclear.

In this study, the differences of longitudinal 2D-STE variables among groups were prominent when the analyses were based upon averaged global deformation comparing to the analyses across six myocardial regions. This might be due the definition of segments. The definition of segment and post-processing software was based on human nomenclature or anatomy, and it may not be necessarily applied to the dogs [31]. Averaged global deformation might reasonably reflect the differences observed among groups over the individual segments.

Limitations of this study include low number of dogs. The dexamethasone suppression test and ACTH concentrations were not applied to make a differential diagnosis of ADH and PDH. HAC of iatrogenic origin was predominant in the area where the authors practice. The differential diagnosis of ADH and PDH was based on the results of the ACTH stimulation test and ultrasonographic images of adrenal glands. Many HAC cases in which bilaterally and irregularly enlarged/round adrenals were presented were excluded from the study. No corresponding aldosterone concentrations were included in this study.
